# Governance structure affects transboundary disease management under alternative objectives

**DOI:** 10.1186/s12889-021-11797-3

**Published:** 2021-10-02

**Authors:** Julie C. Blackwood, Mykhaylo M. Malakhov, Junyan Duan, Jordan J. Pellett, Ishan S. Phadke, Suzanne Lenhart, Charles Sims, Katriona Shea

**Affiliations:** 1 Department of Mathematics and Statistics, Williams College, Williamstown, 01267 MA USA; 2 Division of Biostatistics, School of Public Health, University of Minnesota, Minneapolis, 55455 MN USA; 3 Center for Complex Biological Systems, University of California Irvine, Irvine, 92697 CA USA; 4 Department of Mathematics, University of Tennessee, Knoxville, 37996 TN USA; 5 Department of Economics, University of North Carolina at Chapel Hill, Chapel Hill, 27516 NC USA; 6 Department of Economics, University of Tennessee, Knoxville, 37996 TN USA; 7 Howard H. Baker Jr. Center for Public Policy, University of Tennessee, Knoxville, 37996 TN USA; 8 Department of Biology, The Pennsylvania State University, University Park, Pennsylvania, 16802 USA; 9 Center for Infectious Disease Dynamics, The Pennsylvania State University, University Park, Pennsylvania, 16802 USA

**Keywords:** Governance, Infectious disease, Management, Mathematical model, Migration, Objectives, Public health policy

## Abstract

**Background:**

The development of public health policy is inextricably linked with governance structure. In our increasingly globalized world, human migration and infectious diseases often span multiple administrative jurisdictions that might have different systems of government and divergent management objectives. However, few studies have considered how the allocation of regulatory authority among jurisdictions can affect disease management outcomes.

**Methods:**

Here we evaluate the relative merits of decentralized and centralized management by developing and numerically analyzing a two-jurisdiction *SIRS* model that explicitly incorporates migration. In our model, managers choose between vaccination, isolation, medication, border closure, and a travel ban on infected individuals while aiming to minimize either the number of cases or the number of deaths.

**Results:**

We consider a variety of scenarios and show how optimal strategies differ for decentralized and centralized management levels. We demonstrate that policies formed in the best interest of individual jurisdictions may not achieve global objectives, and identify situations where locally applied interventions can lead to an overall increase in the numbers of cases and deaths.

**Conclusions:**

Our approach underscores the importance of tailoring disease management plans to existing regulatory structures as part of an evidence-based decision framework. Most importantly, we demonstrate that there needs to be a greater consideration of the degree to which governance structure impacts disease outcomes.

**Supplementary Information:**

The online version contains supplementary material available at (10.1186/s12889-021-11797-3).

## Background

In our increasingly globalized world, infectious diseases are rarely confined to a single country or administrative region. The SARS-CoV-2 virus, for example, has crossed national borders and spread to every continent. At more local scales, outbreaks of diseases such as seasonal influenza similarly span multiple cities or government districts. Infectious diseases do not respect political boundaries, yet public health policy decisions are often made at such national or sub-national levels of government. This poses a nontrivial management problem, since different administrative districts and levels of government often have differing objectives, distinct demographics, and inequitable access to health care resources. The impact this can have on public health outcomes is vividly illustrated by the approach to outbreak management for the COVID-19 pandemic in the United States, which was heavily state-devolved at first but has now shifted to a much more federal-level approach.

The scale mismatch between areas of disease prevalence and regulatory jurisdictions leads to a fundamental tradeoff in infectious disease management. Decentralized management allows each local government to tailor management responses to the specifics of its population. For example, a country may differentially prefer travel bans or physical distancing measures based on the amount of migration into the country and its population density. However, decentralized approaches risk coordination failures that result when the disease spreads across borders and might lead to selfish policies in which a governmental agency does not consider how its actions affect neighboring jurisdictions. In contrast, centralized management offers better coordination and can account for movement between regions, but may be unable to tailor responses to the heterogeneous populations impacted by the disease. Centralized management is also complicated by the tendency of lower governmental levels to under-report cases [1] and object to a central power’s decisions. For example, at the beginning of the COVID-19 pandemic several countries applied unilateral travel restrictions despite World Health Organization recommendations to the contrary, and many rural counties in the United States argued that state-imposed mandates on business closures were unnecessary for their communities.

A natural question arises: is it generally better to manage an infectious disease at a decentralized or at a centralized level? Research in mathematical epidemiology has used metapopulation models to illustrate how spatial heterogeneity and varied connectivity between populations affect disease spread [2–7], and some work has employed optimal control theory with time-varying controls to suggest centralized management plans that minimize disease burden and costs [8–11]. However, these studies limit their analyses to a single manager that optimizes a global objective by choosing potentially unrealistic time-varying controls. On the other hand, the law and economics literature has sought to understand how authority for environmental regulation should be allocated between federal, state, and local governments [12–14]. Although such work offers few clear directives, it does highlight an important tradeoff: local governments are more adaptive and have more accurate information about the cost of environmental regulation, while national governments are better equipped to assess and implement regulation for pollutants or populations that cross local political boundaries. Similar ideas have been proposed in public health studies [1, 15]. For example, the lack of coordinated public health policies has been cited in criticism of Canada’s and Australia’s responses to SARS [16, 17] and the United States’ response to bioterrorism [18, 19]. Yet at the same time, centralized responses to public health emergencies are known to underperform policies tailored to local conditions [20, 21], and debates over the proper level of government to manage infectious diseases have recently come to the forefront in light of the COVID-19 pandemic [22–25].

To our knowledge, no study has fully incorporated both aspects of this tradeoff in a comprehensive disease modeling framework. Herein, we attempt to clarify the relative merits of different governance structures by employing a two-patch Susceptible-Infected-Recovered-Susceptible (*SIRS*) model. That is, we adapt the classic *SIR* model paradigm [26, 27] to additionally account for immunity loss. This type of disease model breaks a population into compartments based on each individual’s disease status. Individuals are then able to move between classes based on disease-related processes; for example, a susceptible individual moves into the infected class following interaction with and successful transmission from an infected individual. In our model, each patch represents a spatially distinct population and we allow for migration between these patches. Borders are assumed to be porous in that human movement between them can be transient or permanent. This migration is the key feature that connects the two patches, permits the disease to spread between jurisdictions, and makes the governance issues important. Using this modeling framework, a main objective is to demonstrate that governance structure is an important consideration in designing best strategies for disease mitigation.

We use this modeling framework to achieve two objectives. First, demonstrate the extent to which governance structure is an important consideration in designing best strategies for disease mitigation. While the importance of governance structure is well recognized in other contexts (e.g., pollution control, natural resource management) it is less recognized in the epidemiology and public health literature. Second, to highlight when the differences between local self-interest and global objectives are large. When these differences are small, uniform best management practices and testing protocols will be more likely to be accepted and implemented by local jurisdictions.

In our analysis, we consider three management approaches. A decentralized management scheme allows each patch to unilaterally choose actions that minimize the disease’s impact within its borders, without considering the other patch’s actions or objectives. Centralized management is characterized by a single decision-maker that seeks to minimize the total impact of the disease across both patches, but we distinguish between two possibilities for how the decision-maker chooses actions. Jurisdiction-specific centralized management allows the decision-maker to choose different actions for each patch based on the current state of the disease within each jurisdiction, while uniform management requires the decision-maker to implement a one-size-fits-all solution by choosing the same action for both patches. Using this modeling framework, we establish general recommendations for optimal governance in the presence of the three management types (decentralized, jurisdiction-specific centralized, and uniform centralized) given two standard objectives (to minimize the number of cases or to minimize the number of deaths) and five alternative control methods (vaccination, isolation, medication, border closure, and a travel ban on infected individuals). These types of control methods change the transmission or transition rates in our model. For example, vaccination moves individuals directly from the susceptible (*S*) class to the recovered (*R*) class, isolation moves individuals into the compartment where transmission does not occur, and medication directly impacts the recovery rate of infected individuals. Additionally, border closures and travel bans for infected individuals affect human movement from one patch to the other.

## Methods

To assess whether decentralized or centralized management results in better health outcomes, we treat infectious disease control as a Structured Decision Making problem [28, 29]. First, we outline the two-patch *SIRS* model that we will analyze. Then we define five alternative interventions, each of which can be implemented at different intensity levels. Further, we characterize the differences between decentralized, jurisdiction-specific centralized, and uniform centralized management levels. Our model allows each management level to select from two public health objectives: minimizing the number of infectious cases and minimizing the number of deaths. As such, we evaluate the expected consequences of alternative interventions at various intensities for each level of management given each of the two objectives. Finally, we identify the best management action for each combination of management level and control objective.

### The base model

We consider two spatially distinct populations *A* and *B*, each of which exists in a separate “patch.” If considering the control of a disease spreading within a single country, these patches represent sub-national governments (e.g. states, provinces, or territories). Alternatively, if considering the control of a disease spreading internationally, these patches represent individual countries with susceptible populations. For consistency we will refer to the patches as jurisdictions. We model infectious disease dynamics in each jurisdiction using a canonical frequency-dependent *SIRS* model without demography and use parameters characteristic of a highly infectious disease that has both disease-induced mortality and a relatively rapid rate of immunity loss.

The rate at which susceptible individuals acquire the disease can differ between the jurisdictions. Each jurisdiction has a force of infection given by *λ*_*i*_=*βS*_*i*_*I*_*i*_/*N*_*i*_, where *β* is the transmission rate and *i* denotes the particular jurisdiction (*A* or *B*) so that *N*_*i*_=*S*_*i*_+*I*_*i*_+*R*_*i*_ is the total population size in jurisdiction *i*. Once infected, individuals recover at a baseline rate *γ* and die at a rate *ϕ*. Note that recovered individuals are those who have acquired temporary immunity by either recovering from the disease or receiving a vaccine. Additionally, we assume that individuals lose their immunity and return to the susceptible class at a rate *ζ*. For simplicity, we assume that the transmission rate, the baseline recovery rate, and the rate of immunity loss are inherent to the pathogen itself and hence do not differ between jurisdictions. However, differences in the force of infection across jurisdictions (due to differences in initial conditions) imply that one-size-fits-all control strategies will not always be best.

We want to model porous borders between the two jurisdictions, so we incorporate migration for individuals in all classes. Here, migration refers to both permanent and transient migration. That is, we are not tracking the origin jurisdiction of individuals who move across the border and we therefore do not consider immigration policy in guiding our discussion. However, we recognize that this is an important consideration for future work on the impact of travel bans in mitigating disease spread.

We assume that migration rates are equal for the susceptible and recovered classes, and denote the rate at which *S*-class or *R*-class individuals move from jurisdiction *j* to jurisdiction *i* by *δ*_*ij*_. Infected individuals may migrate at a lower rate due to travel restrictions, so we denote the rate at which *I*-class individuals move from jurisdiction *j* to jurisdiction *i* by *τ*_*ij*_. Note that in the absence of travel restrictions, *δ*_*ij*_=*τ*_*ij*_. These assumptions result in the following two-jurisdiction *SIRS* model:

1 $$ \left\{ \begin{aligned} \frac{dS_{A}}{dt} &= - \beta S_{A} \frac{I_{A}}{N_{A}} + \zeta R_{A} + \delta_{{AB}} S_{B} - \delta_{{BA}} S_{A} \\ \frac{dI_{A}}{dt} &= \beta S_{A} \frac{I_{A}}{N_{A}} - (\gamma + \phi) I_{A} + \tau_{{AB}} I_{B} - \tau_{{BA}} I_{A} \\ \frac{dR_{A}}{dt} &=\gamma I_{A} - \zeta R_{A} + \delta_{{AB}} R_{B} - \delta_{{BA}} R_{A} \\ \end{aligned} \right.  $$

2 $$ \left\{ \begin{aligned} \frac{dS_{B}}{dt} &= - \beta S_{B} \frac{I_{B}}{N_{B}} + \zeta R_{B} + \delta_{{BA}} S_{A} - \delta_{{AB}} S_{B} \\ \frac{dI_{B}}{dt} &= \beta S_{B} \frac{I_{B}}{N_{B}} - (\gamma + \phi) I_{B} + \tau_{{BA}} I_{A} - \tau_{{AB}} I_{B} \\ \frac{dR_{B}}{dt} &=\gamma I_{B} - \zeta R_{B} + \delta_{{BA}} R_{A} - \delta_{{AB}} R_{B} \\ \end{aligned} \right.  $$

Each jurisdiction has an initial population size *N*_*i*_(0)=1,000,000. We consider four different initial conditions: (1) each jurisdiction has a single infectious individual, (2) jurisdiction *A* has a single infectious individual and jurisdiction *B* is undergoing an outbreak (10% of individuals are initially infected), (3) jurisdiction *A* is undergoing an outbreak and jurisdiction *B* has a single infectious individual, and (4) both jurisdictions are undergoing an outbreak. The relevance of including both (2) and (3) becomes clear when considering decentralized management, since results may change depending on which jurisdiction is implementing controls. In all of our analyses, we simulate the model for one year to capture the dynamics of a single epidemic outbreak cycle. All model parameters are provided in Table 1.

**Table 1:** Model parameters

Parameter	Definition	Value
*β*	Transmission rate	3/5
*ϕ*	Disease-induced mortality rate	1/7
*γ*	Baseline recovery rate	1/5
*ζ*	Immunity loss rate	1/270
*δ*_*ij*_	Baseline migration rate from *j* to *i* for susceptible and recovered individuals	0.2
*τ*_*ij*_	Baseline migration rate from *j* to *i* for infected individuals	0.2

All units are in 1/days

### Disease management

We consider five disease control strategies: vaccination, isolation, administration of medication, travel restrictions on all individuals (hereafter referred to as “border closure”), and travel restrictions only on infected individuals (hereafter referred to as a “travel ban”). For simplicity, each jurisdiction can choose to either implement one control strategy or take no action for the duration of the simulation. While control strategies can differ between jurisdictions, both controls are implemented at the same intervention intensity *α*∈[0,1], where *α* is a proportion of the model parameter of interest. *α*=0 indicates that no control is implemented while *α*=1 denotes maximum implementation intensity.

The details of each control strategy are provided below. In the presence of management, the original model equations are modified as follows:

3 $$ {}<InlineMediaObject>
<ImageObject FileRef="12889_2021_11797_Figa_HTML.gif" Format="GIF" Color="BlackWhite" Type="Linedraw" Rendition="HTML" Width="0" Height="0" Resolution="0"/>
<ImageObject FileRef="12889_2021_11797_Figa_Print.eps" Format="EPS" Color="BlackWhite" Type="Linedraw" Rendition="Print"/></InlineMediaObject>  $$

4 $$ {}<InlineMediaObject>
<ImageObject FileRef="12889_2021_11797_Figb_HTML.gif" Format="GIF" Color="BlackWhite" Type="Linedraw" Rendition="HTML" Width="0" Height="0" Resolution="0"/>
<ImageObject FileRef="12889_2021_11797_Figb_Print.eps" Format="EPS" Color="BlackWhite" Type="Linedraw" Rendition="Print"/></InlineMediaObject>  $$

where *L*_*i*_ denotes isolated individuals in jurisdiction *i*. Parameters that can be impacted by management actions implemented in jurisdiction *A* are colored red, while parameters that can be impacted by management actions implemented in jurisdiction *B* are colored blue. Changes to the base model due to disease management are detailed below and in Table 2.

**Table 2:** Control rates and intensity ranges

Control	Definition	Range
$\hat {\gamma }_{i}$	Recovery rate	[1/5, 1]
*η*_*i*_	Proportion of newly infectious individuals in jurisdiction *i* that get isolated	[0, 1]
*ω*_*i*_	Vaccination rate for jurisdiction *i*	[0, 1]
$\hat {\delta }_{{ij}}$	Migration rate from *j* to *i* for susceptible and recovered individuals	[0, 0.2]
$\hat {\tau }_{{ij}}$	Migration rate from *j* to *i* for infected individuals	[0, 0.2]

All rates have units 1/days

#### Vaccination

We consider vaccination in the form of a six month long immunization program. If vaccination is implemented by jurisdiction *i*, then susceptible individuals move to the recovered class at a rate *ω*_*i*_ throughout the entire simulation. We assume that both naturally-acquired and vaccine-derived immunity wane at the same rate *ζ*. In our model, intervention intensity directly scales the average time to vaccination so that

5 $$ \omega_{i} = \frac{2}{365(1-\alpha)}  $$

when 0<*α*<1. When *α*=0, there is no vaccination and therefore *ω*_*i*_=0. When *α*=1 we assume that the susceptible population is vaccinated almost immediately (1 day) so that *ω*_*i*_=1. Further, we assume that when *α*>0 vaccination is rolled out so that all susceptible individuals are vaccinated within six months on average (so that the smallest positive value of *ω*_*i*_ is 2/365).

#### Isolation

To incorporate isolation as a control strategy, we expand the base model by adding an isolation class (*L*_*i*_) to each jurisdiction. Throughout the course of the simulation, a proportion *η*_*i*_ of infected individuals in jurisdiction *i* are moved into isolation. Once in isolation, individuals do not contribute to the force of infection and are not permitted to travel. Consequently, individuals in *L*_*i*_ are also not included in *N*_*i*_. We assume, however, that isolated individuals do not receive special treatment and hence have the same recovery and death rates as infected individuals. In our model intervention intensity directly determines the isolation rate, so that

6 $$ \eta_{i} = \alpha.  $$

#### Medication

We assume that medication may be provided to infected individuals, which has an ultimate effect of increasing the recovery rate. In particular, we assume that the average amount of time spent in the infectious class can be decreased from 5 days to 1 day after medication is provided. To implement medication in jurisdiction *i* at a control intensity *α*, our model linearly scales the recovery rate *γ*_*i*_ by *α* so that

7 $$ \hat{\gamma}_{i} = \gamma (4 \alpha + 1).  $$

#### Border closure

When a jurisdiction implements border closure, it reduces the number of individuals who can migrate from the other jurisdiction. Border closures are implemented uniformly across all disease classes, e.g. migration of susceptible individuals is restricted in the same way as migration of recovered individuals. When *α*=1, a complete border closure is in place and no individuals are allowed to move into the jurisdiction implementing the closure. Note that border closure places no restrictions on outgoing migration: individuals from the jurisdiction implementing the closure can still leave and travel to the other jurisdiction (unless the other jurisdiction is also implementing a border closure policy). In our model, intervention intensity directly determines the reduction in migration, so that

8 $$ \hat{\tau}_{{ij}} = \tau_{{ij}} (1 - \alpha)  $$

and

9 $$ \hat{\delta}_{{ij}} = \delta_{{ij}} (1 - \alpha).  $$

#### Infected travel ban

While border closure hinders travel of individuals in all disease classes of a given jurisdiction, we assume that a travel ban only limits the migration of infected individuals. As in Eq. 8, a travel ban implemented by jurisdiction *i* with intensity *α* will result in

10 $$ \hat{\tau}_{{ij}} = \tau_{{ij}} (1 - \alpha).  $$

However, in contrast to Eq. 9, for travel bans we assume that

11 $$ \hat{\delta}_{{ij}} = \delta_{{ij}}.  $$

### Governance structures

We assume that all levels of government have access to the same set of information and the same set of control strategies. That is, each manager can implement any of the five intervention types at the same intervention intensity as the other jurisdiction. Additionally, we do not allow for collaboration between decentralized management authorities or between centralized and decentralized management authorities when determining control implementation.

#### Decentralized management

When disease management is coordinated at the decentralized level, each jurisdiction chooses a control strategy that most effectively meets its own objective without considering the strategy or objective of the other jurisdiction. In other words, decentralized managers act selfishly. Note that chosen control strategies and management objectives can differ between the two jurisdictions. Since the jurisdictions disregard each other’s status, each jurisdiction makes optimal decisions under the assumption that the other jurisdiction will not implement any control strategy. Realistically, a jurisdiction might assume that a neighboring country or state will free-ride on its own management or may not have the resources to manage the disease.

#### Centralized management

While a decentralized decision maker only has authority to implement control strategies within its own jurisdiction, a centralized decision maker has authority to collectively manage the disease in both jurisdictions at once. The centralized manager seeks to minimize collective epidemic burdens for the total population by choosing a control strategy which most effectively meets that collective objective.

We distinguish between two types of centralized governance structures. A jurisdiction-specific centralized manager may take advantage of jurisdictional differences and implement different interventions in each jurisdiction. For example, this form of management is exhibited when a federal government implements a travel ban in one state and a quarantine in another. A uniform centralized manager, on the other hand, must implement the same control strategy in both jurisdictions. In real-world scenarios, governments exercise uniform centralized management when they are unwilling or unable to tailor disease responses to individual regions and opt for a one-size-fits-all approach.

By definition, jurisdiction-specific centralized management will always outperform both decentralized management and uniform centralized management regardless of the objective. Our analysis, then, lies in considering the additional cases or deaths produced from the other two governance levels. The additional cases or deaths resulting from decentralized management represent the cost of uncoordinated, decentralized control of the disease. Similarly, the additional cases or deaths resulting from uniform centralized management represent the cost of implementing one-size-fits-all public health responses. The relative performance of decentralized management versus uniform centralized management is, in turn, determined by the difference in additional cases or deaths resulting from management by these two governance structures.

### Management objectives

We assume that managers can choose to optimize one of two objectives: the cumulative number of infectious cases or the cumulative number of disease-induced deaths. Although it may seem intuitive to assume that minimizing the number of cases is equivalent to minimizing the number of deaths, this is not necessarily true. Previous work has shown that the number of infectious cases is not a constant scalar multiple of the number of disease-induced deaths [30]. In our model, this occurs because some individuals might get infected multiple times due to immunity loss but each individual can only die once. Therefore, it is necessary to distinguish between these two objectives and consider both separately.

## Results

### Decentralized management

Under the assumption that only jurisdiction *A* applies interventions, the best type of control at the decentralized level depends on both the initial conditions and control intensity (see Table 3). When there is initially a single case in each jurisdiction, it is almost always preferable to administer vaccination. Vaccination is also the preferred management option across all initial conditions when the vaccine is rapidly rolled out.

**Table 3:** Control rankings for decentralized management

Initial condition, objective	Control intensity			
	0.25	0.5	0.75	1
Single case AB, min cases	VMLTCN	VMLCredNT	VMLCredNT	VMLCredNT
Single case AB, min deaths	MVLTCN	VMLTCN	VMLCredNT	VMLCredNT
Outbreak B, min cases	MLVredNCT	MLVredNCT	MLVredNCT	VMLredNTC
Outbreak B, min deaths	MLVredNCT	MLVredNCT	MLVredNCT	VMLredNTC
Outbreak A, min cases	MLVredNCT	MLVredNCT	MLVredNCT	VMLredNCT
Outbreak A, min deaths	MLVredNCT	MLVredNCT	MLVredNTC	VMLredNCT
Outbreak A & B, min cases	MLVredNCT	MLVredNCT	MLVredNCT	VMLredNCT
Outbreak A & B, min deaths	MLVredNCT	MLVredNCT	MLVredNCT	VMLredNCT

Rankings of control types at four different control intensities and four sets of initial conditions (rankings are in order of most effective to least effective). N = no control, V = vaccination, L = isolation, M = medication, C = border closure, and T = travel ban for infected individuals only. Red text reflects scenarios in which no control outperforms a management option

In contrast, when one or both jurisdictions is undergoing an outbreak and the control intensity is imperfect, then medication is the preferred treatment option. Moving individuals quickly into the recovered class following infection minimizes their ability to spread infection to others. Vaccination, on the other hand, is only the better option when it rapidly moves individuals into the recovered class and outpaces the effects of medication.

**Fig. 1:**
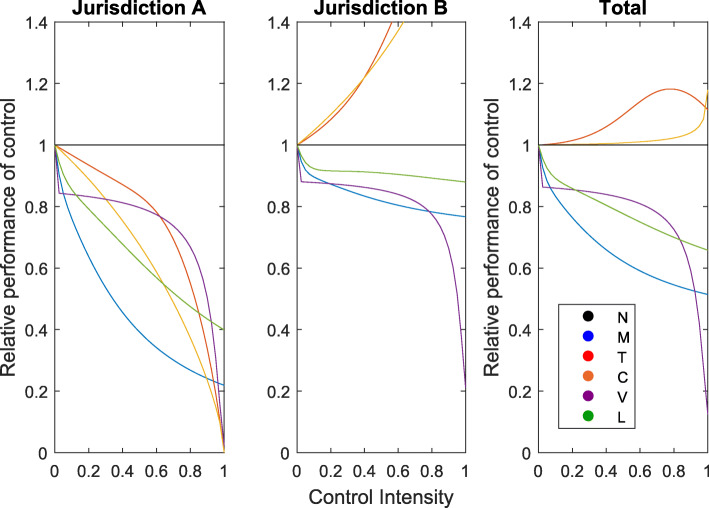
Results for decentralized management when jurisdiction *A* implements the specified control while jurisdiction *B* does not implement any control. Each plot displays the number of cases with a given control applied relative to the number of cases in the absence of control for jurisdiction *A* (left panel), jurisdiction *B* (middle panel), and the total across both jurisdictions (right panel) against control intensity. N = no control, M = medication, T = travel ban for infected individuals only, C = border closure, V = vaccination, and L = isolation

In most of the scenarios we considered, border closures and travel bans actually increase the cumulative number of deaths and cases across both jurisdictions relative to not implementing any control. For example, given an initial condition of 1 infected individual in *A* and 10% of the population infected in *B*, a border closure or travel ban implemented by jurisdiction *A* will decrease the numbers of cases and deaths in jurisdiction *A* but cause an even greater increase in these numbers for jurisdiction *B* (Fig. 1). Thus, one jurisdiction acting in its own best interest may negatively impact the other jurisdiction as well as the overall case load and death toll. These results align with findings from [31]. The only exception results from an initial condition of a single infectious individual in each jurisdiction. In this case, a partial border closure or travel ban can often mitigate disease impacts relative to no control (see Table 3). However, it is never as successful as medication, vaccination, or isolation.

Increasing the intensity of a control tends to amplify the general trends outlined above. In particular, increasing the control intensity for a vaccination, medication, or isolation program decreases the number of cases and the number of deaths (Fig. 1). Further graphical representations of our results can be found in the Supplementary Information (SI).

### Centralized management

Our results demonstrate that management practices which were the best choices for decentralized governance might no longer be optimal when the disease is managed at a centralized level. Moreover, the effectiveness of centralized control depends on whether the management authority is limited to uniform, one-size-fits-all policies or is able to tailor control strategies to each jurisdiction and thereby take advantage of jurisdictional differences in the force of infection.

#### Uniform centralized management

When there is a single infectious case in each jurisdiction, the relative performance of vaccination, isolation, and medication depends on the intensity at which the control is applied. At low intensity, vaccination is preferred. For higher control intensities, on the other hand, all three control options are nearly equally effective under uniform centralized management. Other initial conditions, however, result in objective-dependent changes to the rankings of management options (Table 4).

**Table 4:** Control rankings for uniform centralized management

Initial condition, objective	Control intensity			
	0.25	0.5	0.75	1
Single case AB, min cases	VML, T=C=N	M=L, V, T=C=N	M=L, V, T=C=N	V=M=L, T=C=N
Single case AB, min deaths	VML, T=C=N	M=L, V, T=C=N	M=L, V, T=C=N	V=M=L, T=C=N
Outbreak B, min cases	MLVredNTC	MLVredNTC	MLVredNTC	VMLCredNT
Outbreak B, min deaths	MLVredNTC	MLVredNTC	MLVredNTC	MLVCredNT
Outbreak A, min cases	MLVredNTC	MLVredNTC	MLVredNTC	VMLCredNT
Outbreak A, min deaths	MLVredNTC	MLVredNTC	MLVredNTC	MLVCredNT
Outbreak A & B, min cases	MLV, N=T=C	MLV, N=T=C	MLV, N=T=C	VML, N=T=C
Outbreak A & B, min deaths	MLV, N=T=C	MLV, N=T=C	MLV, N=T=C	MLV, C=N=T

Rankings of control types at four different control intensities and four sets of initial conditions (rankings are in order of most effective to least effective). N = no control, V = vaccination, L = isolation, M = medication, C = travel ban for all individuals, and T = travel ban for infected individuals only. Red text reflects scenarios in which no control outperforms a management option

For all initial conditions where at least one jurisdiction is already undergoing an outbreak, treating infected individuals with medication is usually more effective than vaccination. This observation, however, depends critically on the management objective: vaccination outperforms medication if the control intensity is sufficiently high and the objective is to minimize the number of cases, not deaths. Intuitively, in such a scenario a medication program can prevent more deaths than a vaccine. The total number of cases, however, would be greater than under a strategy of vaccination. This result contrasts with the case of decentralized management, where vaccination is preferred under both objectives if the control intensity is sufficiently high. In the decentralized scenario, only one jurisdiction is being managed. If the objective is to minimize the number of deaths and medication is applied in one jurisdiction, then dispersal of infected individuals is capable of causing enough cases (and subsequent deaths) to suggest vaccination as a more effective management scenario. Of course, these results apply for our model and parameterization but, in practice, will depend on the particular dispersal and disease-specific dynamics.

As with decentralized management, full border closures or travel bans under a uniform centralized government are capable of increasing the numbers of cases and deaths relative to no control. Under some conditions (e.g. a single infection in each jurisdiction), implementing a full border closure or travel ban does not reduce cases or deaths relative to the no-control scenario. Occasionally, there is a positive effect of full border closure but the positive impact of this strategy on meeting objectives is negligible relative to vaccination, isolation, and medication. Graphical representations of these results are provided in the SI.

#### Jurisdiction-specific centralized management

Given an initial condition of one case in each jurisdiction, vaccinating one jurisdiction while administering medication in the other is the best management strategy at low control intensities. Administering medication in both jurisdictions is the best option as intervention intensities increase. An exception is that vaccination is preferred in both jurisdictions if the objective is to minimize the number of cases and control intensity is sufficiently high.

When the objective is to minimize the number of infectious cases and one or both jurisdictions is undergoing an outbreak, the results for jurisdiction-specific centralized management align with those for uniform centralized management (Table 5). In particular, administering medication in both jurisdictions is the best management policy for lower control intensities and switches to vaccination when the control intensity is high. The results are also consistent across governance structures under the same conditions when the objective is to minimize the number of deaths, with exception of very high control intensity.

**Table 5:** Control rankings for jurisdiction-specific centralized management

Initial condition, objective	Control intensity			
	0.25	0.5	0.75	1
Single case AB, min cases	V, M	M, M	M, M	V, V
Single case AB, min deaths	V, M	M, M	M, M	M, M
Outbreak B, min cases	M, M	M, M	M, M	V, V
Outbreak B, min deaths	M, M	M, M	M, M	C, M
Outbreak A, min cases	M, M	M, M	M, M	V, V
Outbreak A, min deaths	M, M	M, M	M, M	M, C
Outbreak A & B, min cases	M, M	M, M	M, M	V, V
Outbreak A & B, min deaths	M, M	M, M	M, M	M, M

The best control strategy for jurisdiction *A* (listed first) and jurisdiction *B* (listed second) at four different control intensities and four sets of initial conditions. N = no control, V = vaccination, L = isolation, M = medication, C = border closure, and T = travel ban for infected individuals only

Interestingly, in the case of high control intensity and an objective of minimizing the number of deaths, jurisdiction-specific border closure can be optimal when one jurisdiction begins mid-outbreak while the other initially has a single infectious individual. Administering medication to the jurisdiction undergoing an outbreak while implementing border closure for the other jurisdiction will be the best course of action. This suggests that border closures may be most effective in preventing outbreaks from establishing in jurisdictions with few infected individuals.

**Fig. 2:**
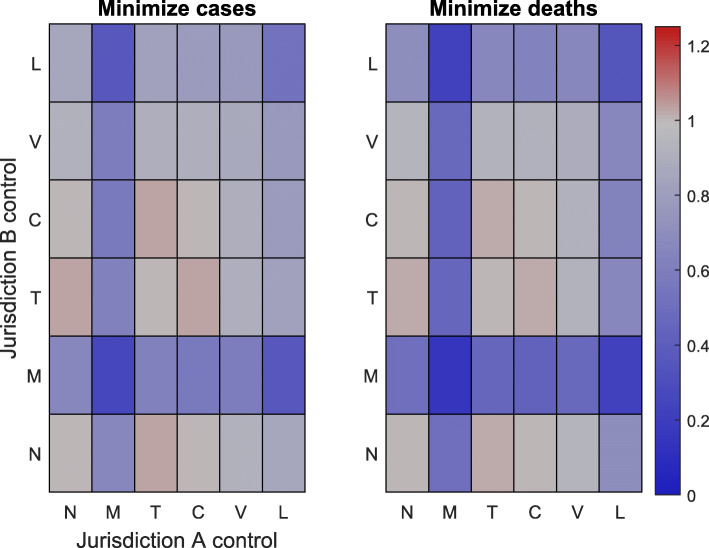
Results for centralized management when different types of control are applied in each jurisdiction. Here both jurisdictions are initially in an outbreak state (10% of each population is initially infected). Each square corresponds to a different combination of control options, with jurisdiction *A* represented on the horizontal axis and jurisdiction *B* represented on the vertical axis. The colors on the left (right) grid denote the total number of cases (deaths) across both jurisdictions relative to the total number of cases (deaths) in the absence of control. Gray indicates that the combination of controls does not change the outcome relative to the no-control scenario and red (blue) indicates that there are more (fewer) cases or deaths relative to the no-control scenario

**Fig. 3:**
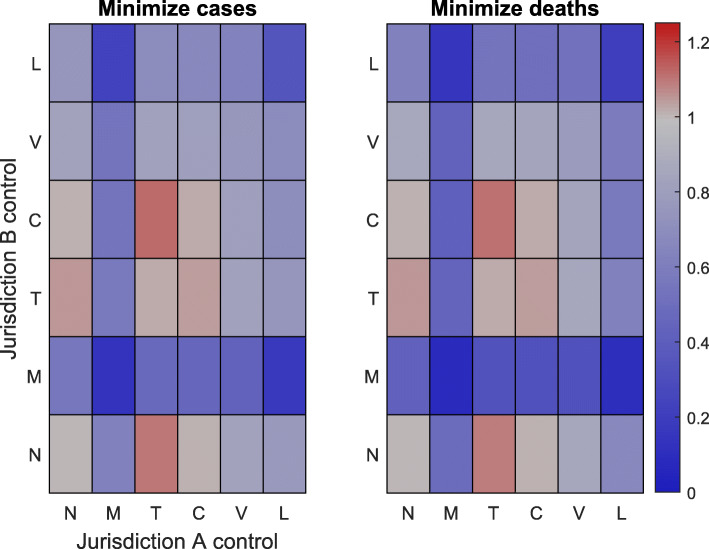
Results for centralized management when different types of control are applied in each jurisdiction. Here jurisdiction *B* is initially in an outbreak state (10% of the population is initially infected) whereas jurisdiction *A* initially has a single infected individual. Each square corresponds to a different combination of control options, with jurisdiction *A* represented on the horizontal axis and jurisdiction *B* represented on the vertical axis. The colors on the left (right) grid denote the total number of cases (deaths) across both jurisdictions relative to the total number of cases (deaths) in the absence of control. Gray indicates that the combination of controls does not change the outcome relative to the no-control scenario and red (blue) indicates that there are more (fewer) cases or deaths relative to the no-control scenario

**Fig. 4:**
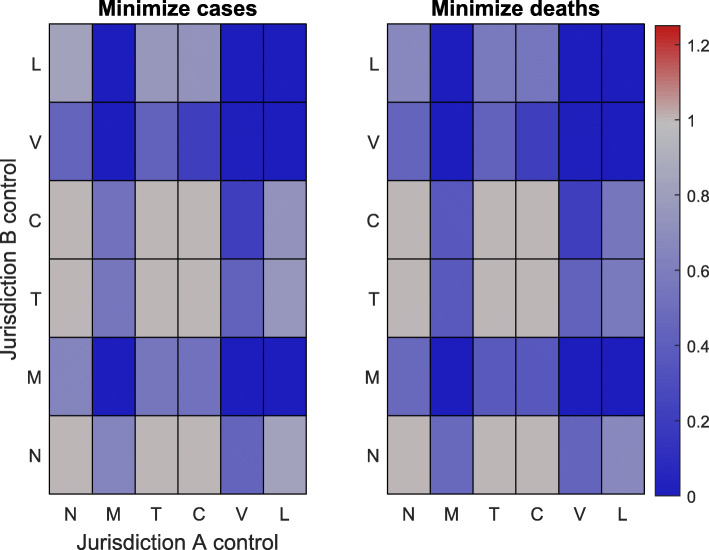
Results for centralized management when different types of control are applied in each jurisdiction. Here each of the two jurisdictions initially has a single infected individual. Each square corresponds to a different combination of control options, with jurisdiction *A* represented on the horizontal axis and jurisdiction *B* represented on the vertical axis. The colors on the left (right) grid denote the total number of cases (deaths) across both jurisdictions relative to the total number of cases (deaths) in the absence of control. Gray indicates that the combination of controls does not change the outcome relative to the no-control scenario and red (blue) indicates that there are more (fewer) cases or deaths relative to the no-control scenario

Simulation results for all possible combinations of interventions at every initial condition are provided in Figs. 2, 3 and 4 under a control intensity of *α*=0.5. Note that the simulation results for an initial outbreak only in jurisdiction *A* are provided in the SI, as these results are the transpose of Fig. 3. In all cases, any management policy that uses either vaccination, isolation, or medication in at least one jurisdiction leads to an overall reduction in both the number of cases and the number of deaths relative to no control.

### When does local self-interest achieve global objectives?

A key result we wish to highlight is that policies formed in the best interest of individual jurisdictions may not achieve global objectives. Table 6 shows the best management strategy for jurisdiction *A* from a decentralized perspective (first entry) and two possible global perspectives (centralized one-size-fits-all, second entry; centralized jurisdiction-specific, third entry). Bracketed entries indicate that the centralized decision maker is indifferent between the indicated controls. Here, we assume the centralized decision maker will use jurisdiction *A*’s preference as a “tie breaker,” to avoid conflicts between local and central governments. Orange entries indicate scenarios where the best interest of jurisdiction A will not achieve the global objective. Cyan entries indicate situations where disagreements between centralized and decentralized decision makers may arise depending on whether the centralized decision maker utilizes one-size-fits-all or jurisdiction-specific controls.

**Table 6:** Best controls for jurisdiction *A* under each governance structure

Initial condition, objective	Control intensity			
	0.25	0.5	0.75	1
Single case AB, min cases	V, V, [V, M]	orangeV, [orangeM, orangeL], orangeM	orangeV, [orangeM, orangeL], orangeM	blackV, [blackV, blackM, blackL], blackV
Single case AB, min deaths	cyanM, cyanV, [cyanV, cyanM]	orangeV, [orangeM, orangeL], orangeM	orangeV, [orangeM, orangeL], orangeM	orangeV, [orangeV, orangeM, orangeL], orangeM
Outbreak B, min cases	M, M, M	M, M, M	M, M, M	V, V, V
Outbreak B, min deaths	M, M, M	M, M, M	M, M, M	orangeV, orangeM, [orangeC, orangeM]
Outbreak A, min cases	M, M, M	M, M, M	M, M, M	V, V, V
Outbreak A, min deaths	M, M, M	M, M, M	M, M, M	orangeV, orangeM, [orangeM, orangeC]
Outbreak A & B, min cases	M, M, M	M, M, M	M, M, M	V, V, V
Outbreak A & B, min deaths	M, M, M	M, M, M	M, M, M	orangeV, orangeM, orangeM

The first entry in each list of controls is the choice of a decentralized manager, the second entry is the choice of a uniform centralized manager, and the third entry is the choice of a jurisdiction-specific centralized manager. Orange entries indicate scenarios where the best interest of individual jurisdictions will not achieve the global objective. Cyan entries indicate scenarios where the best interest of individual jurisdictions may not achieve global objectives

If one jurisdiction is undergoing an outbreak, then the policy that is in the best interest of jurisdiction *A* is usually also chosen by both types of centralized management. Thus, if the centralized and decentralized decision makers’ objective is to minimize cases, local self-interest will generally achieve global objectives. An exception, however, is if the control intensity is sufficiently high and the objective is to minimize the number of deaths. In such cases, the policy in the best interest of jurisdiction *A* (vaccination) is not as successful as the global approach of applying medication or a combination of medication and travel restrictions. In the latter case, the global objective is better achieved by implementing a complete travel ban to prevent disease establishment in one jurisdiction while managing the other with medication.

However, if there is only a single case in each jurisdiction then policies that are in the best interest of jurisdiction *A* may not achieve global objectives regardless of the control intensity. When jurisdiction *A* is free to choose the control type that best mitigates the impacts of disease within its borders, it almost always chooses vaccination in our particular disease parameterization (with the exception of low control intensity and an objective of minimizing deaths). A centralized manager, however, will often instead prescribe medication to jurisdiction *A*.

## Conclusions

Public health policy is often complicated by the fact that political boundaries rarely correspond to the boundaries of populations and risk factors. This mismatch is especially pertinent when managing an infectious disease that impacts multiple regulatory jurisdictions [32]. Governments in the various jurisdictions might have different management objectives, unequal access to resources, and disparate views on cooperation. And no matter how distinct these political divisions might seem, the movement of individuals between jurisdictions indicates that actions taken by the management authority in one jurisdiction will affect disease dynamics in neighboring jurisdictions as well. Thus, policymakers must carefully consider what level of governance is ideal for managing infectious diseases in a transboundary setting.

We developed and analyzed a mathematical model to assess the benefits and drawbacks of different governance structures in a two-jurisdiction setting. In particular, we considered how each of three management levels would choose from among five control strategies when seeking to minimize either the number of cases or the number of deaths. Our results demonstrate that control outcomes can strongly depend on two governance factors: (1) whether control is implemented at a centralized or decentralized level and (2) whether the managers’ objectives are to minimize cases or minimize deaths.

In general, we find that disease management policies created in the best interest of individual jurisdictions may not be optimal from a global perspective. Under the conditions presented in this work, this can arise when managers seek to minimize the number of disease-induced deaths. Given this objective, a centralized manager may prescribe medication when a decentralized manager would instead choose to vaccinate. This is especially true when the intensity of the vaccine roll-out is high. Moreover, our results illustrate how jurisdiction-specific centralized management outperforms uniform centralized management. A centralized government with the ability to act equitably *in terms of health outcomes* will always outperform a government that acts equitably *in terms of control efforts*. Similar results have also been shown by studies of measles vaccination efforts in Malawi, where vaccination strategies that focused on generating equity in outcomes yielded better results than the strategy of equally administering vaccines to all regions [20, 21].

Under certain scenarios, we find that decentralized management policies can even increase the overall numbers of cases or deaths. This occurs in our model for both types of travel restrictions: full border closure and a travel ban on infected individuals. Note that the negative impact of travel bans is much less pronounced under centralized management, which suggests that the negative impact of travel restrictions depends on whether they are implemented at a centralized or decentralized level. Under jurisdiction-specific centralized management, such travel restrictions can be the best management strategy for a jurisdiction that is disease-free while using another strategy in a jurisdiction undergoing an outbreak. This implies that such restrictions might be most effective for preventing new outbreaks rather than controlling existing outbreaks across multiple regions. The result that travel interventions can actually worsen disease outbreaks is echoed in the theoretical work of Hsieh et al. (2007) [31]. In real world situations, travel restrictions have led to unintended consequences in addition to directly exacerbating case and death numbers. For example, travel restrictions imposed by the United States and European countries during the 2014 West African Ebola outbreak did curb spread to those countries, but the commercial flights that did not leave West Africa also did not return. As a result, transporting medical equipment, supplies, and personnel back into the outbreak region became more difficult.

In terms of the relative merits of control strategies themselves, our results suggest that allocating effort into developing and administering effective vaccinations and medications should be prioritized. In contrast, focusing effort on travel bans may only be most effective when attempting to prevent the initial establishment of a disease in a given region. However, it is important to note that our results are based on a single parameterization of a coupled two-patch *SIRS* model and are thus parameter-dependent. For example, we show in the SI that negative impacts of travel bans are diminished for lower rates of immunity loss. We also evaluate our results on a one-year time scale, whereas other timescales may be more relevant in other scenarios. A more thorough consideration of uncertainty is certainly warranted in future research, and methods exist that examine uncertainty in projections about the impact of management interventions [29, 33–35]. Rather than attempt to quantify the possible range of uncertainty, we have opted to present a set of examples that clearly demonstrate how governance structure and choice of objectives can have crucial implications in the implementation of strategies for disease management.

We also did not assign economic costs to the control strategies. While vaccination may be the best strategy to minimize cases or deaths, it might be more expensive than other strategies. These costs should be considered when making actual disease management decisions. However, modeling the costs of interventions would have presented several challenges that are beyond the scope of the current paper. First, incorporating disease- and country-specific costs would have limited our ability to make general points about the importance of governance structure. Second, a model with costs must be analyzed under a cost-effectiveness framework, where preferred control strategies are those that minimize the cost of limiting the number of cases or deaths to a given level. In order to implement this approach, judgments must be made about the acceptable numbers of cases or deaths. Further research that considers economic, political, and social factors is certainly needed, but we do not anticipate that accounting for these factors would change our results concerning the relative advantages of centralized and decentralized disease management. Relatedly, it may be important for future models to allow for even greater flexibility in governance structure depending on specific real-world scenarios.

In summary, we have provided a modeling framework that can be used to examine how different governance structures across multiple jurisdictions can affect disease management outcomes in a wide variety of settings. Although we present theoretical examples, our approach can be tailored to any focal disease management situation and relevant governance structures. We showed how to assess the success or failure of alternative control strategies and, more significantly, we demonstrated that those outcomes depend on the choice of governance level and management objectives. Our results indicate that in certain situations uniform, centralized control outperforms a decentralized approach, provided such policy is guided by scientific advice on optimal control strategies. However, there are also situations where federal authorities can achieve better outcomes by delegating decisions to the regional or local level, thereby allowing more finely-tuned responses to local conditions. Ultimately, our results accentuate a critical need for states and countries to cooperate on ensuring equitable outcomes when managing infectious disease outbreaks [36–40].

## Supplementary Information

**Additional file 1Supplemental Information**. Governance structure affects transboundary disease management under alternative objectives.

## Data Availability

Not applicable.
