# Supplementary material for: Governance structure affects transboundary disease management under alternative objectives
Source: BMC Public Health. 2021 Oct 2;21:1782. doi: 10.1186/s12889-021-11797-3 (PMC8487237; doi:10.1186/s12889-021-11797-3)
Supplement: Supplementary file 1 — Additional file 1Supplemental Information. Governance structure affects transboundary disease management under alternative objectives. [file 12889_2021_11797_MOESM1_ESM.pdf]

Supplemental Information: Governance structure affects transboundary disease management under alternative objectives

Julie C Blackwood, Mykhaylo M. Malakhov, Junyan Duan, Jordan J. Pellett, Ishan Phadke, Suzanne Lenhart, Charles Sims, and Katriona Shea

Supplemental Information

Lifelong immunity

In this section, we reproduce Tables 3–6 as well as Figure 1 from the main text when immunity is lifelong (Tables 1–4 and Figure 1 here). The primary difference from the main results is that travel bans and border closures are much less likely to generate an overall increase in cases (or deaths) under our parameterization.

Vaccination relative to medication are almost always ranked the same as in the main text. Because immunity is lifelong, there are fewer overall cases and/or deaths in the case of no control (since reinfection is not possible). Therefore, the best strategy is whichever (medication or vaccination) moves individuals into the recovered classes the fastest.

Table 1: Control rankings for decentralized management with lifelong immunity

Initial condition, objective	Control intensity			
	0.25	0.5	0.75	1
Single case AB, min cases	VMLTCN	VMLCTN	VMLTCN	VMLTCN
Single case AB, min deaths	VMLTCN	VMLTCN	VMLTCN	VMLTCN
Outbreak B, min cases	MLVTCN	MLVTCN	MLVTCN	VMLTCN
Outbreak B, min deaths	MLVTCN	MLVTCN	MLVTCN	VMLTCN
Outbreak A, min cases	MLVTCN	MLVTCN	MLVTCN	VMLTCN
Outbreak A, min deaths	MLVTCN	MLVTCN	MLVTCN	VMLTCN
Outbreak A & B, min cases	MLVTCN	MLVTCN	MLVTCN	VMLTCN
Outbreak A & B, min deaths	MLVTCN	MLVTCN	MLVTCN	VMLTCN

Table 1: Rankings of control types at four different control intensities and four sets of initial conditions (rankings are in order of most effective to least effective). N = no control, V = vaccination, L = isolation, M = medication, C = border closure, and T = travel ban for infected individuals only. Red text reflects scenarios in which no control outperforms a management option.

Table 2: Control rankings for uniform centralized management with lifelong immunity

Initial condition, objective	Control intensity			
	0.25	0.5	0.75	1
Single case AB, min cases	VML, T=C=N	M=L, V, T=C=N	M=L, V, T=C=N	V=M=L, T=C=N
Single case AB, min deaths	VML, T=C=N	M=L, V, T=C=N	M=L, V, T=C=N	V=M=L, T=C=N
Outbreak B, min cases	MLVCTN	MLVCTN	MLVCTN	VMLTNC
Outbreak B, min deaths	MLVCTN	MLVCTN	MLVCTN	MLVTNC
Outbreak A, min cases	MLVCTN	MLVCTN	MLVCTN	VMLTNC
Outbreak A, min deaths	MLVCTN	MLVCTN	MLVCTN	MLVTNC
Outbreak A & B, min cases	MLV, T=C=N	MLV, T=C=N	MLV, T=C=N	VML, T=C=N
Outbreak A & B, min deaths	MLV, T=C=N	MLV, T=C=N	MLV, T=C=N	MLV, T=C=N

Table 2: Rankings of control types at four different control intensities and four sets of initial conditions (rankings are in order of most effective to least effective). N = no control, V = vaccination, L = isolation, M = medication, C = travel ban for all individuals, and T = travel ban for infected individuals only. Red text reflects scenarios in which no control outperforms a management option.

Table 3: Control rankings for jurisdiction-specific centralized management with lifelong immunity

Initial condition, objective	Control intensity			
	0.25	0.5	0.75	1
Single case AB, min cases	V, M	M, M	M, M	V, V
Single case AB, min deaths	V, M	M, M	M, M	M, M
Outbreak B, min cases	M, M	M, M	M, M	V, V
Outbreak B, min deaths	M, M	M, M	M, M	C, M
Outbreak A, min cases	M, M	M, M	M, M	V, V
Outbreak A, min deaths	M, M	M, M	M, M	M, C
Outbreak A & B, min cases	M, M	M, M	M, M	V, V
Outbreak A & B, min deaths	M, M	M, M	M, M	M, M

Table 3: The best control strategy for jurisdiction A (listed first) and jurisdiction B (listed second) at four different control intensities and four sets of initial conditions. N = no control, V = vaccination, L = isolation, M = medication, C = border closure, and T = travel ban for infected individuals only.

Table 4: Best controls for jurisdiction A under each governance structure with lifelong immunity

Initial condition, objective	Control intensity			
	0.25	0.5	0.75	1
Single case AB, min cases	V, V, [V, M]	V, [M, L], M	V, [M, L], M	V, [V, M, L], V
Single case AB, min deaths	V, V, [V, M]	V, [M, L], M	V, [M, L], M	V, [V, M, L], M
Outbreak B, min cases	M, M, M	M, M, M	M, M, M	V, V, V
Outbreak B, min deaths	M, M, M	M, M, M	M, M, M	V, M, [C, M]
Outbreak A, min cases	M, M, M	M, M, M	M, M, M	V, V, V
Outbreak A, min deaths	M, M, M	M, M, M	M, M, M	V, M, [M, C]
Outbreak A & B, min cases	M, M, M	M, M, M	M, M, M	V, V, V
Outbreak A & B, min deaths	M, M, M	M, M, M	M, M, M	V, M, M

Table 4: The first entry in each list of controls is the choice of a decentralized manager, the second entry is the choice of a uniform centralized manager, and the third entry is the choice of a jurisdiction-specific centralized manager. Orange entries indicate scenarios where the best interest of individual jurisdictions will not achieve the global objective. Cyan entries indicate scenarios where the best interest of individual jurisdictions may not achieve global objectives.

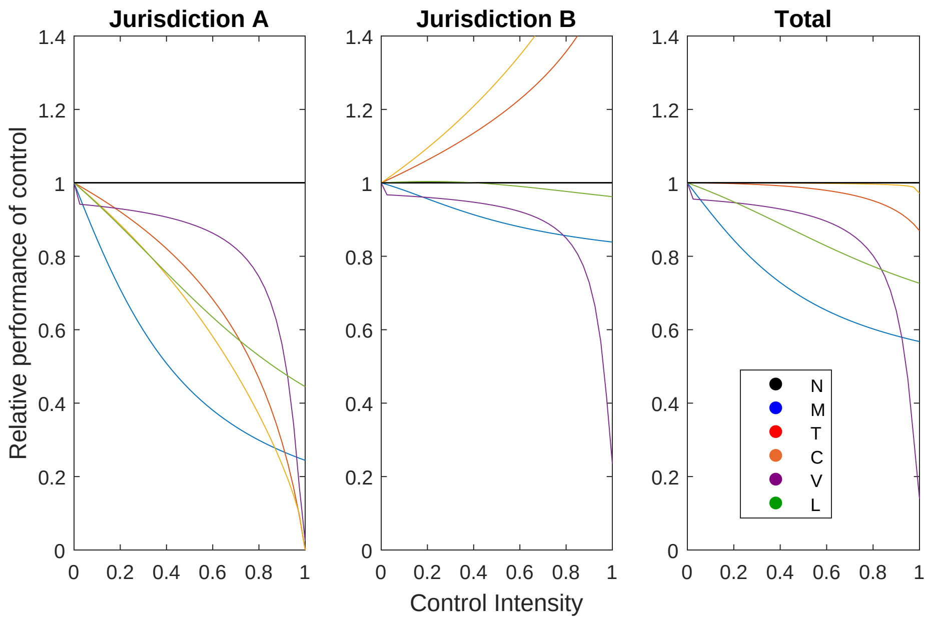

Figure 1: Results for decentralized management only such that jurisdiction A implements the specified control for each control intensity whereas jurisdiction B implements no control. This figure shows the number of cases with a given control applied relative to the number of cases in the absence of control for jurisdiction A (left panel), jurisdiction B (middle panel), and the total across both jurisdictions (right panel). Each control is displayed with a different color. Here, yellow and red represent the travel ban and border closure, respectively. In contrast to Figure 1 in the main text, immunity is lifelong

Variation of initial conditions

Included in this section are our results for decentralized and centralized control for sets of initial conditions that are not included in the main paper.

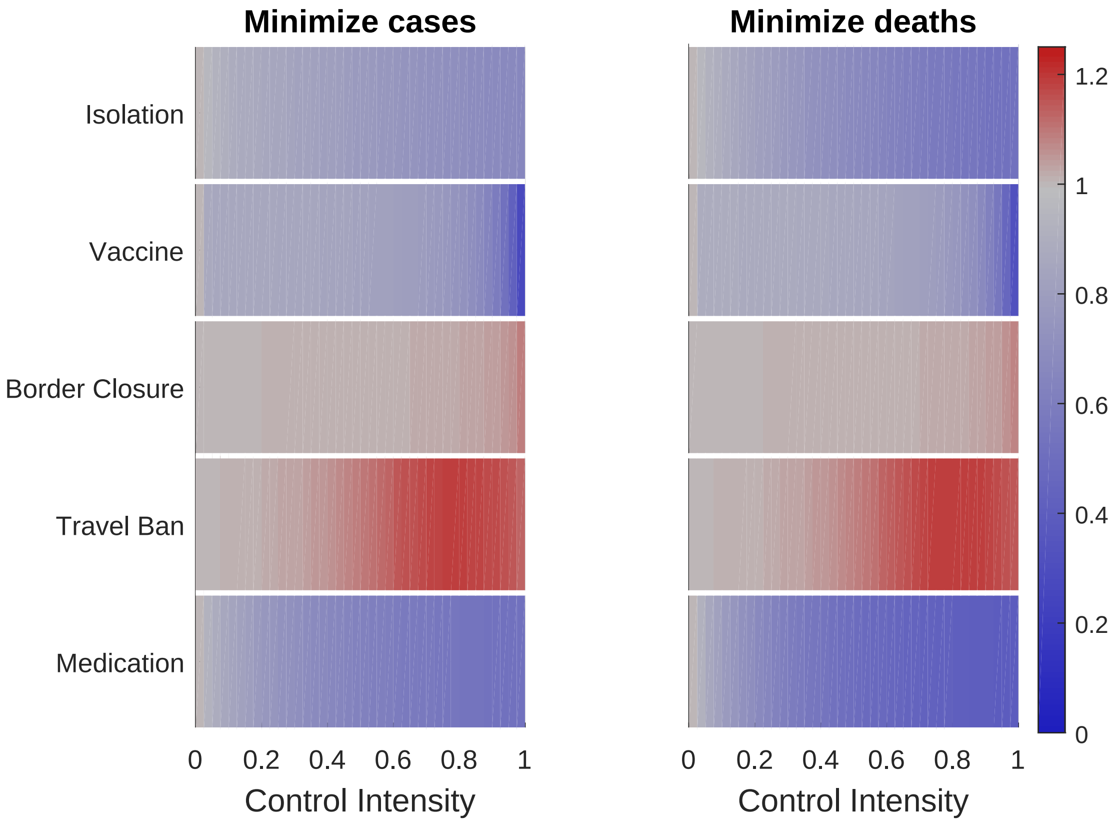

Figure 2: Results for decentralized management only such that jurisdiction A implements the specified control for each control intensity whereas jurisdiction B implements no control. In this scenario, jurisdiction B is initially in an outbreak state (10% of each population is initially infected) whereas jurisdiction A has only a single infection present. The colors in the left (right) column demonstrate the total number of cases (deaths) across both jurisdictions relative to the total number of cases (deaths) in the absence of control. A value of 1 (gray) indicates that a given control does not change the outcome relative to the no control scenario. Red (blue) indicates that there are more (fewer) cases or deaths relative to the no control scenario.

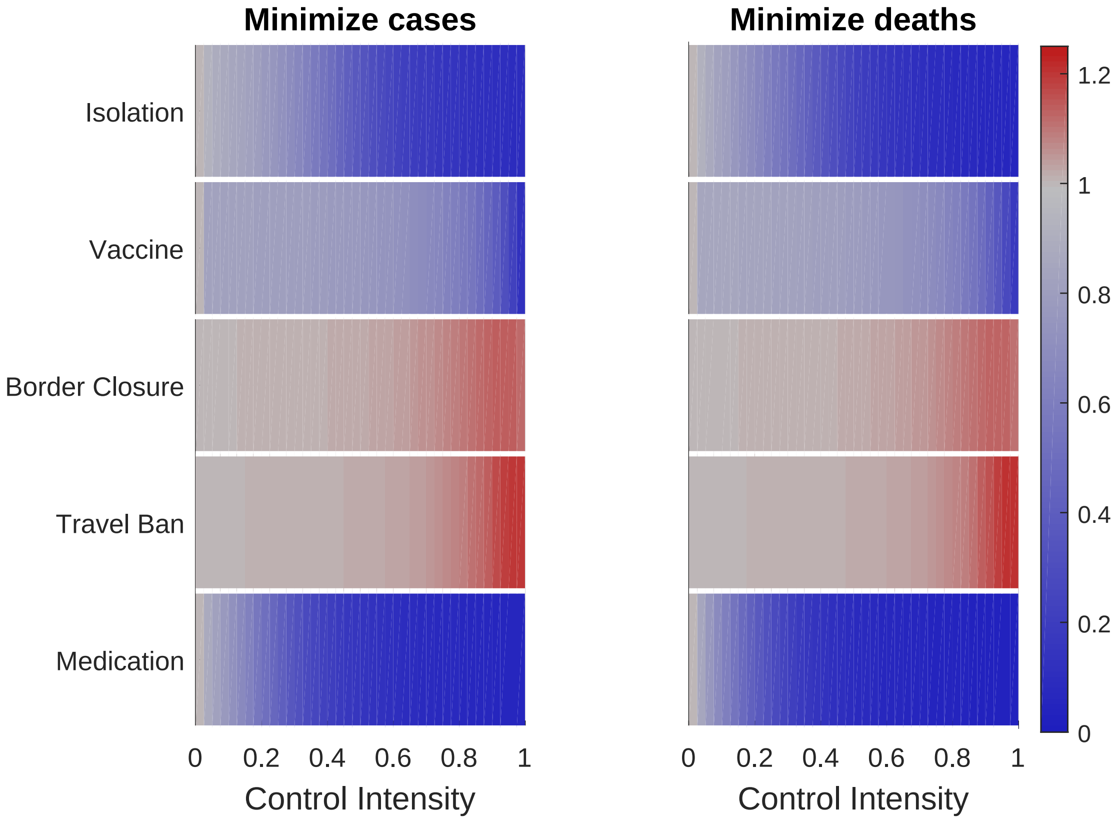

Figure 3: Results for uniform centralized management where the same type and intensity of control are applied in both jurisdictions. In this scenario, jurisdiction B is initially in an outbreak state (10% of each population is initially infected) whereas jurisdiction A has only a single infection present. The colors in the left (right) column demonstrate the total number of cases (deaths) across both jurisdictions relative to the total number of cases (deaths) in the absence of control. A value of 1 (gray) indicates that a given control does not change the outcome relative to the no control scenario. Red (blue) indicates that there are more (fewer) cases or deaths relative to the no control scenario.

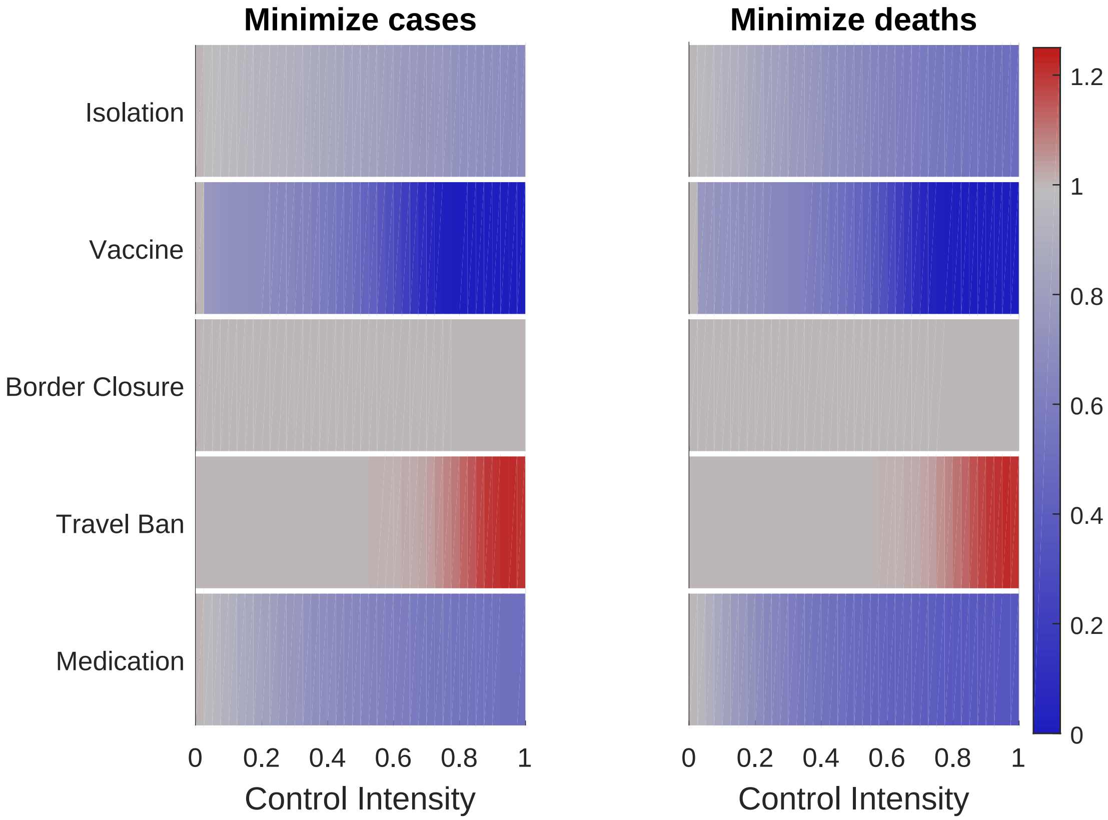

Figure 4: Results for decentralized management only such that jurisdiction A implements the specified control for each control intensity whereas jurisdiction B implements no control. In this scenario, both jurisdictions initially only have a single infected individual. The colors in the left (right) column demonstrate the total number of cases (deaths) across both jurisdictions relative to the total number of cases (deaths) in the absence of control. A value of 1 (gray) indicates that a given control does not change the outcome relative to the no control scenario. Red (blue) indicates that there are more (fewer) cases or deaths relative to the no control scenario.

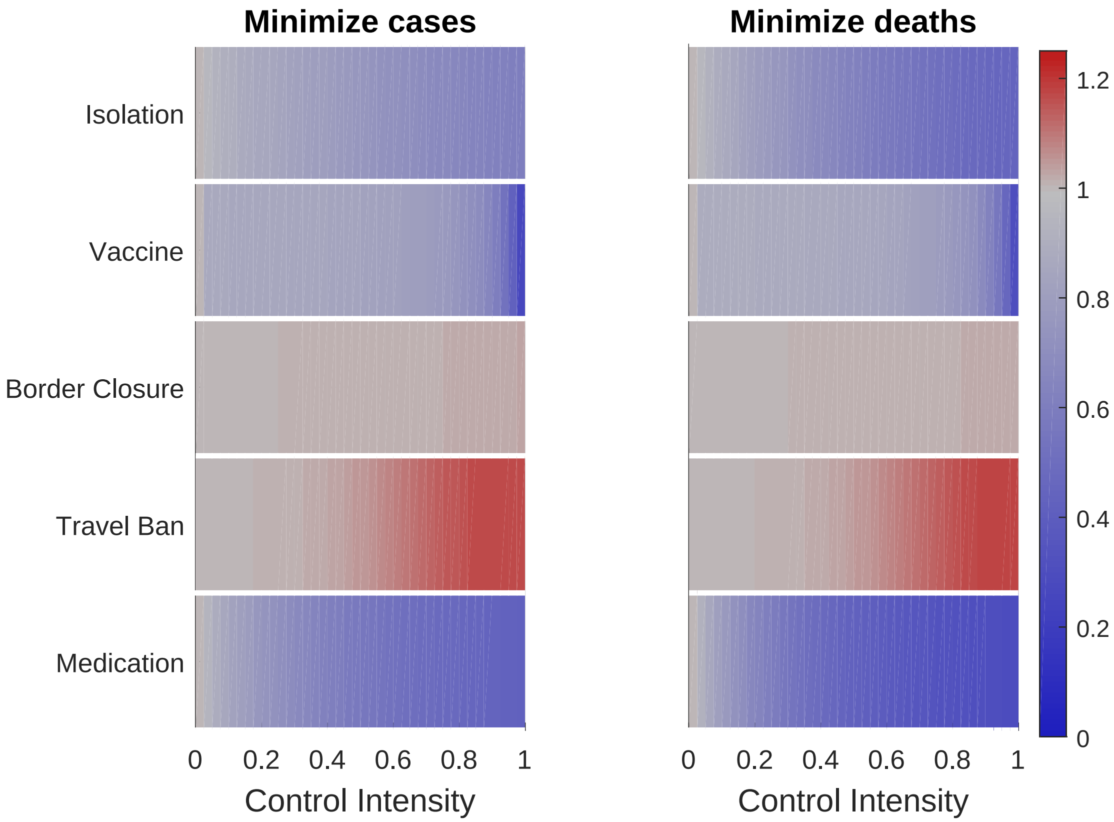

Figure 5: Results for decentralized management only such that jurisdiction A implements the specified control for each control intensity whereas jurisdiction B implements no control. In this scenario, jurisdiction A is initially in an outbreak state (10% of each population is initially infected) whereas jurisdiction B has only a single infection present. The colors in the left (right) column demonstrate the total number of cases (deaths) across both jurisdictions relative to the total number of cases (deaths) in the absence of control. A value of 1 (gray) indicates that a given control does not change the outcome relative to the no control scenario. Red (blue) indicates that there are more (fewer) cases or deaths relative to the no control scenario.

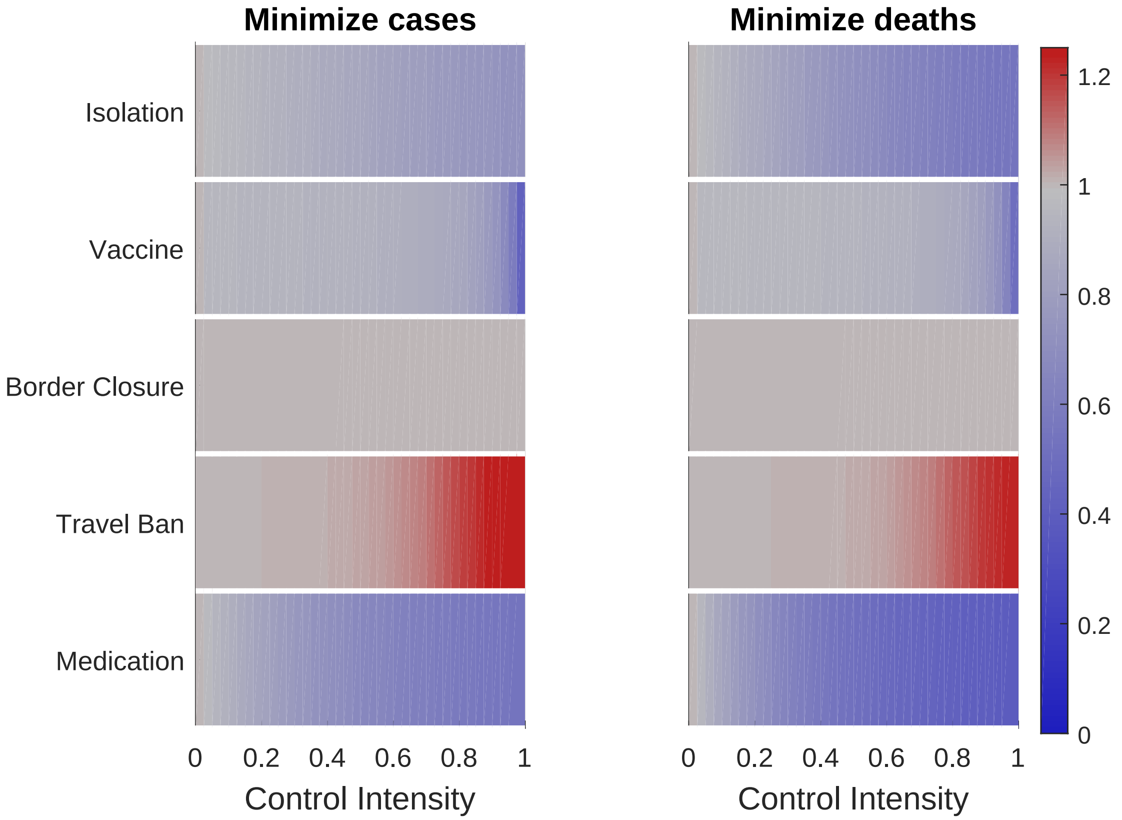

Figure 6: Results for decentralized management only such that jurisdiction A implements the specified control for each control intensity whereas jurisdiction B implements no control. In this scenario, both jurisdictions are initially in an outbreak state (10% of each population is initially infected). The colors in the left (right) column demonstrate the total number of cases (deaths) across both jurisdictions relative to the total number of cases (deaths) in the absence of control. A value of 1 (gray) indicates that a given control does not change the outcome relative to the no control scenario. Red (blue) indicates that there are more (fewer) cases or deaths relative to the no control scenario.

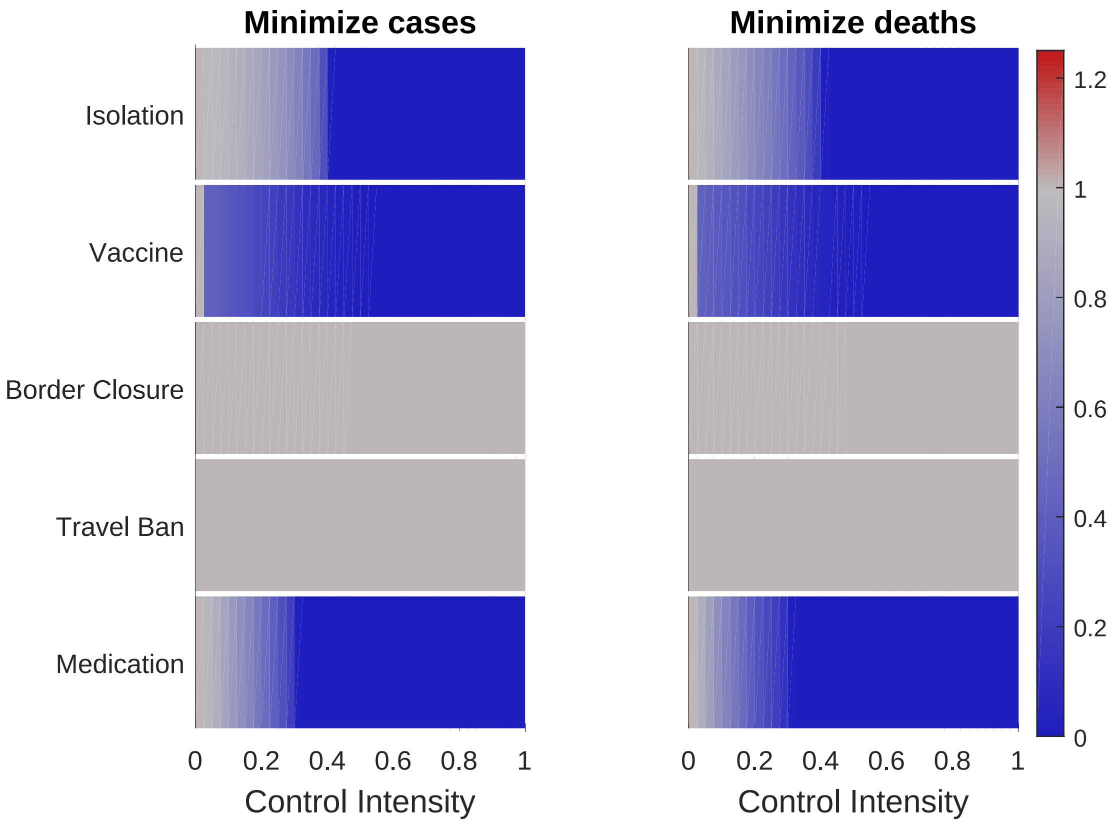

Figure 7: Results for uniform centralized management only such that both jurisdictions apply the same type and level of control. In this scenario, both jurisdictions initially have only a single infected individual. The left column displays the total number of cases across both jurisdictions relative to the total number of cases in the absence of control. The right column displays the total number of deaths across both jurisdictions relative to the total number of deaths in the absence of control.

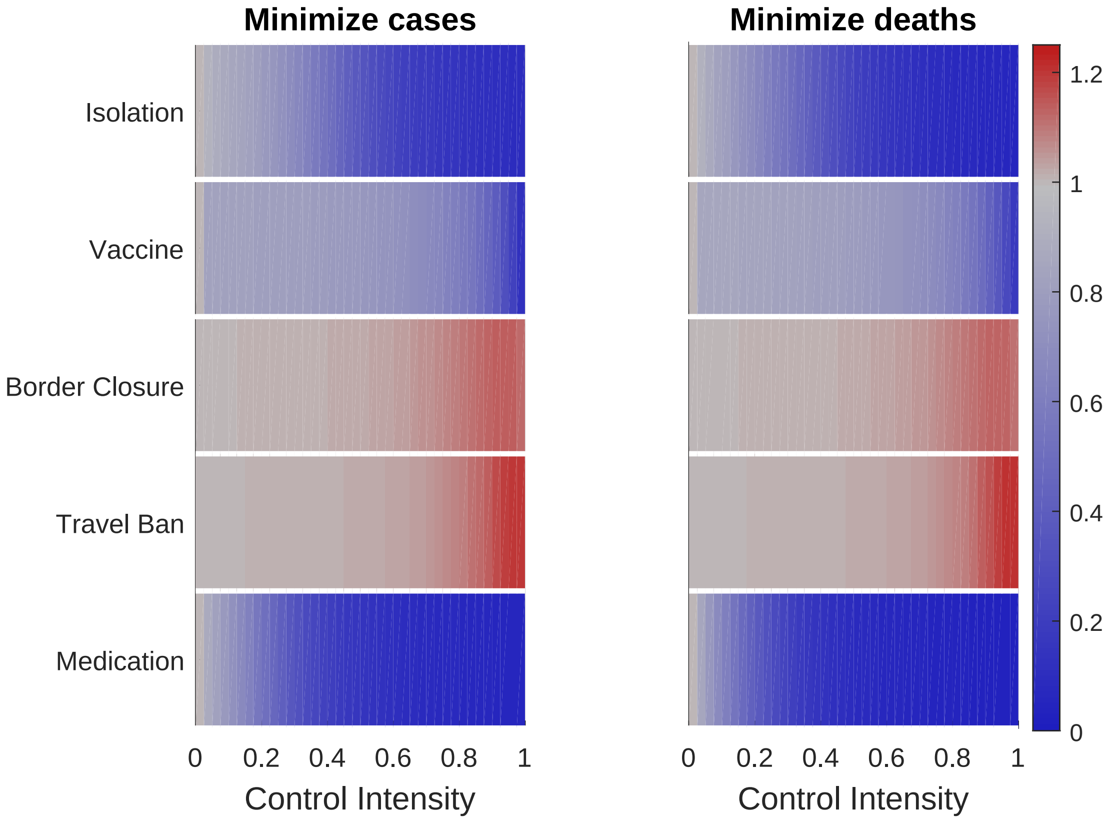

Figure 8: Results for uniform centralized management only such that both jurisdictions apply the same type and level of control. In this scenario, jurisdiction A is initially in an outbreak state (10% of each population is initially infected) whereas jurisdiction B has only a single infection present. The colors in the left (right) column demonstrate the total number of cases (deaths) across both jurisdictions relative to the total number of cases (deaths) in the absence of control. A value of 1 (gray) indicates that a given control does not change the outcome relative to the no control scenario. Red (blue) indicates that there are more (fewer) cases or deaths relative to the no control scenario.

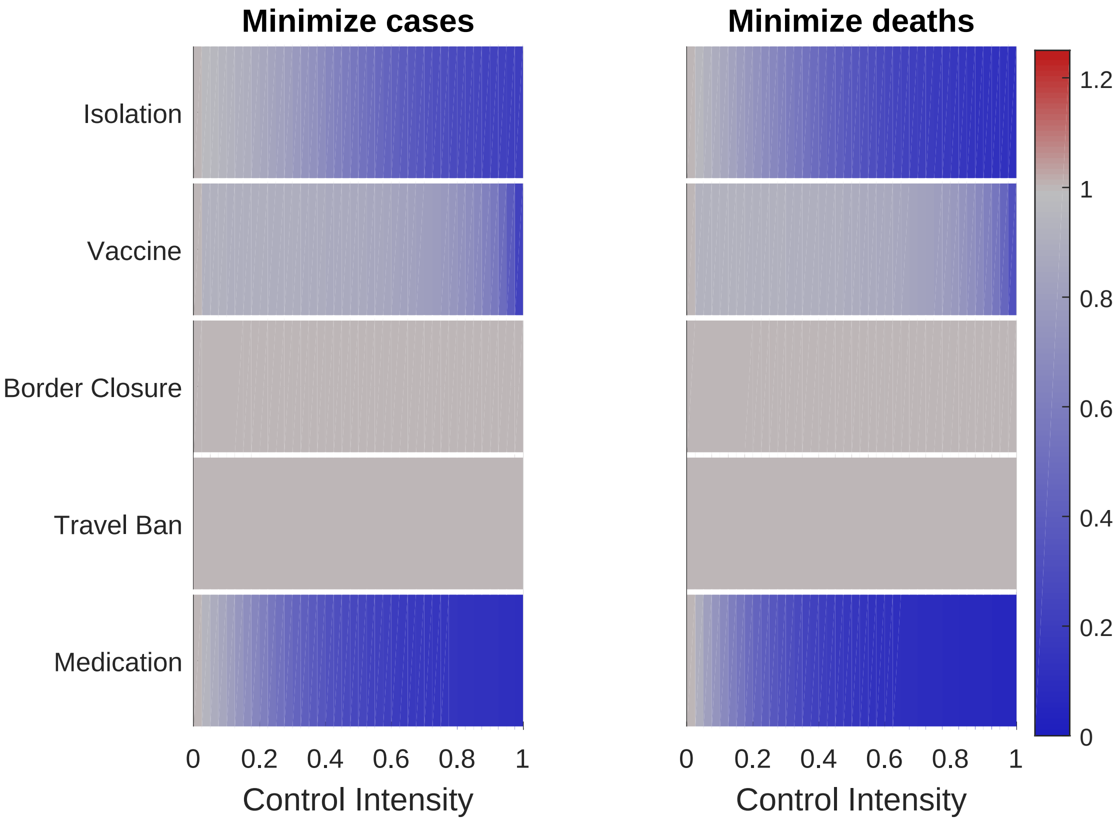

Figure 9: Results for uniform centralized management only such that both jurisdictions apply the same type and level of control. In this scenario, both jurisdictions are initially in an outbreak state (10% of each population is initially infected). The colors in the left (right) column demonstrate the total number of cases (deaths) across both jurisdictions relative to the total number of cases (deaths) in the absence of control. A value of 1 (gray) indicates that a given control does not change the outcome relative to the no control scenario. Red (blue) indicates that there are more (fewer) cases or deaths relative to the no control scenario.

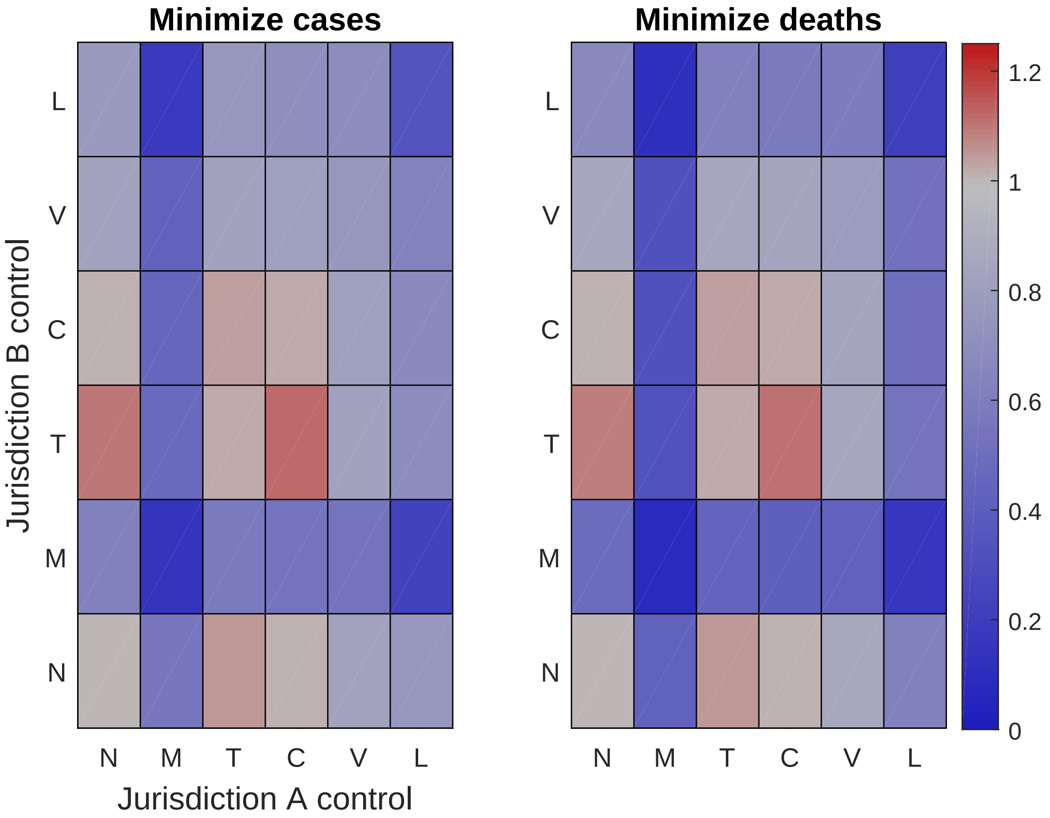

Figure 10: Results for a jurisdiction-specific centralized management such that each jurisdiction can apply a different type of control but at the same intensity. In this scenario, jurisdiction A is initially in an outbreak state (10% of each population is initially infected) whereas jurisdiction B has only a single infection present. The colors in the left (right) column demonstrate the total number of cases (deaths) across both jurisdictions relative to the total number of cases (deaths) in the absence of control. A value of 1 (gray) indicates that a given control does not change the outcome relative to the no control scenario. Red (blue) indicates that there are more (fewer) cases or deaths relative to the no control scenario.
